# Relational attitudes in adolescent girls with and without a diagnosis of anorexia nervosa or atypical anorexia nervosa

**DOI:** 10.1186/s40337-023-00896-8

**Published:** 2023-09-22

**Authors:** Rachel Bachner-Melman, Roni Rom, Lilac Lev-Ari, Iris Shachar-Lavie, Orit Krispin, Rami Tolmacz

**Affiliations:** 1 Clinical Psychology Graduate Program, Ruppin Academic Center, Emek Hefer, Israel; 2 School of Social Work, Hebrew University of Jerusalem, Jerusalem, Israel; 3 Baruch Ivcher School of Psychology, Reichman University, Herzliya, Israel; 4 The Lior Tsfaty Center for Suicide and Mental Pain Studies, Ruppin Academic Center, Emek Hefer, Israel; 5 Child and Adolescent Psychiatry, Schneider Children’s Medical Center of Israel, Petah Tikva, Israel

**Keywords:** Relational attitudes, Anorexia nervosa, Atypical anorexia nervosa, Sense of relational entitlement, Pathological concern

## Abstract

**Background:**

People with eating disorders experience interpersonal difficulties, but little research explores relational attitudes in this population. We examined sense of relational entitlement towards parents, pathological concern, and psychological distress in adolescent girls with and without anorexia nervosa (AN) or atypical anorexia nervosa (AAN).

**Methods:**

Questionnaires assessing sense of entitlement towards parents, pathological concern, and symptoms of depression and anxiety were completed by 85 girls with and 100 girls without AN/AAN (mean age 15.06 ± 1.41). The AN/AAN group also completed a measure of ED pathology.

**Results:**

Eating pathology, pathological concern and symptoms of depression and anxiety were positively associated with both restricted and inflated sense of entitlement towards parents. AN/AAN participants scored significantly higher than controls on restricted and inflated sense of entitlement, pathological concern and symptoms of depression and anxiety. Restricted sense of entitlement and pathological concern partially mediation the association between AN/AAN and symptoms of depression and fully mediated the association between AN/AAN and anxiety. Within the AN/AAN group, pathological concern and symptoms of depression explained a large proportion of the variance in ED pathology.

**Conclusions:**

Adolescent AN/AAN takes a heavy toll on emotional and social health, perhaps in part because crucial aspects of relational mutuality fail to develop. Teens with AN/AAN tend to over-focus on their parents’ needs at the expense of their own needs. They also have impaired capacity to realistically appraise expectations from their parents, tending to feel over- and/or under-entitled to need fulfillment. These relational attitudes are associated with symptoms of depression and anxiety and should be addressed in therapy.

## Abstract (plain language summary)

In healthy relationships people trust another person to fulfill some but not all their needs. It is maladaptive to expect the fulfillment of all needs (inflated entitlement) or of no needs (restricted entitlement). Restricted entitlement generally masks frustration and an unconscious wish for need fulfillment, so that restricted and inflated entitlement can co-occur. We examined to what degree 85 adolescent girls with a restricting ED and 100 without an ED felt entitled to need fulfillment by their parents, and to what extent they sacrificed their own needs for their parents’. Girls with an ED reported higher levels of both restricted and inflated entitlement from their parents than the other girls, and reported placing their parents’ needs before their own more often. The more restricted and/or inflated their entitlement was, the more they tended to place their parents’ needs before their own, to have severe ED symptoms and to feel depressed and anxious. Teens with EDs may over-focus on their parents’ needs at the expense of their own and feel they deserve to have all or none of their needs fulfilled by their parents. These relational attitudes are associated with symptoms of depression and anxiety and should be addressed in therapy.

## Introduction

Eating disorders (EDs) are multifactorial psychiatric illnesses with serious physiological, cognitive, emotional, and interpersonal consequences [57]. There has recently been a significant rise in their prevalence, especially during the COVID-19 pandemic [40]. Age of onset is typically during adolescence [58], when peer relationships are paramount, romantic relationships emerge, and emotional and social support is sought less exclusively from parents. Parents nevertheless remain significant attachment figures, especially in times of stress [1]. The relationship between adolescents and their parents is therefore of potential interest in the context of EDs.

Parent-adolescent relationships are intricately intertwined with developmental trajectories [19] and changes in the ways in which teens’ adjustment is influenced by the parent–child relationship [45]. A recent narrative review of relational issues in adolescents’ EDs emphasized the relevance of relational factors to the development and maintenance of EDs [21]. Results of this review supported the importance of family functioning in EDs, and in particular of parent-adolescent relationships. Unresolved conflict between adolescents’ simultaneous need for autonomy and for support from parents was also found to be associated with EDs [23]. Clarification about mechanisms underlying the relational difficulties of adolescents with EDs would be useful, theoretically and clinically.

Adolescents with EDs may tend either to expect others, like their parents, to treat them with unrealistic sensitivity [2] or to relinquish and neglect their own needs in the service of others [38]. Tolmacz et al. 53, 54 found that the dissatisfaction that adult women from a community sample with high scores on a measure of disordered eating experience from relationships with their romantic partners is partly explained by imbalanced sense of relational entitlement, pathological concern and inauthenticity. Even though these women did not have clinical eating disorders, results suggest a possible link between EDs and problematic relational attitudes. This study examines sense of relational entitlement and pathological concern in a sample of adolescents with anorexia nervosa and atypical anorexia nervosa (AAN), focusing on their relationships with their parents.

Since parent-adolescent conflict can appear on a backdrop of relational imbalance and psychological distress [16], we speculated that imbalanced relational attitudes of adolescents with EDs towards their parents may partly underly their psychological distress in some way, by contributing to their distress and/or resulting from it. We therefore examined sense of relational entitlement towards parents, pathological concern and psychological distress (depression and anxiety) in adolescents with and without a diagnosis of AN or AAN (AN without low weight despite significant weight loss [27]. Eating disorders have consistently been shown to have strong associations with both depression [15] and anxiety [42], and we hypothesized that the relational variables examined in this study (sense of relational entitlement and pathological concern) may contribute to an understanding of this association, i.e., play a mediating role.

### Pathological concern

Pathological concern [43, 49] involves compulsive concern for others in parallel to denial of one’s own feelings and needs. In intersubjective terms, it develops when mutual recognition is lacking so that the self is experienced from an early age as an object and the other as a subject. This kind of concern is characterized by a combination of repression and denial of one’s own needs, and an overinvestment in satisfying others’ [43].

Although concern is usually considered a positive trait, it has pathological expressions [7] when accompanied by a lack of self-regard and over-caring for others [26]. Excessive caring has been viewed as a means of coping with unsatisfied needs [10] and narcissistic vulnerability [36]. Placing others’ needs before one’s own may feel immediately gratifying but is associated with distress and subjective emptiness [52], poor mental health [7], insecure attachment, low self-worth, negativity, and low life satisfaction [43]. People with high pathological concern report inauthenticity in their relationships and dissatisfaction from them [53, 54].

According to the theory of self-psychology, women with EDs feel guilty when looking after their own needs and avoid guilt by over-attending to others’ interests [28]. Research supports the tendency of people with EDs to suppress their own interests, desires, opinions and needs in favor of those of others. ED patients tend to report a particularly submissive, non-assertive interpersonal style [17, 31]. Geller et al. [25] found that AN patients attend to others’ needs and suppress negative feelings to preserve relationships. Brunton et al. [14] found that restrictive eating attitudes and low BMI were associated with the tendency to place others’ needs before one’s own. Bachner-Melman et al., [6] found that women with AN placed others’ needs before their own to a greater degree than control women and that this “selflessness” was associated with ED symptoms in a non-clinical sample. It has also been argued that developmental, interpersonal, family, cultural, genetic, personality and social factors interact to make “pathological altruism” a characteristic of people with EDs [3]. The concept of pathological concern therefore seems particularly pertinent to adolescent girls with EDs.

### Sense of relational entitlement

Traditionally, sense of relational entitlement, or the subjective perception of what one deserves in a relationship [56], has been understood as a pathological, narcissistic trait [20, 39]. More recently, however, it has come to be seen as a universal part of our internal working models [48, 59]. Tolmacz [48] suggested that infants acquire their sense of entitlement via interactions with attachment figures and that it manifests itself in relationships throughout the lifespan [12]. A healthy, or assertive sense of entitlement reflects a balance in the sense of mutuality within relationships,the capacity to recognize the needs of a significant other, without negating one’s own [48]. Sense of relational entitlement becomes inflated when people expect unconditional, total need fulfillment from others, and restricted when people view their needs as illegitimate and do not express them [33]. When one or both of these related forms of entitlement are elevated, sense of relational entitlement is said to be imbalanced, in contrast to a balanced, assertive or healthy sense of entitlement, reflected in low levels of both dimensions [48]. Sense of relational entitlement has been conceptualized in terms of attachment theory [48] and is measured in a parallel way as attachment. Measures of sense of relational sense of entitlement tap two different problematic dimensions (restriction and inflation), just as the Experiences in Close Relationships scale (ECR, [11], widely used to assess attachment style, taps two different problematic dimensions of attachment (anxiety and avoidance). Anxious and avoidant attachment can co-occur (as in disorganized attachment) and low levels in both indicate healthy or secure attachment. In the same way, restricted and inflated sense of relational entitlement can co-occur and low levels of both indicate healthy, assertive or balanced sense of relational entitlement.

Tolmacz et al. [51] proposed the “Sense of Relational Entitlement among Adolescents toward their Parents” scale (SRE-ap) to assess adolescents’ sense of entitlement towards their parents. Using the SRE-ap, imbalanced sense of entitlement was found to be positively associated with insecure attachment, depression, anxiety and school avoidance, and negatively with self-esteem, positive mood and life satisfaction. Most of these variables are correlates of EDs [30]. EDs typically develop during adolescence [58] and often involve interpersonal disturbances [31]. On the one hand, adolescents with AN or AAN require intensive and even exhausting levels of care (need fulfillment) from their parents. On the other hand, paradoxically, they deny and do not acknowledge their own needs [6]. Sense of relational entitlement towards parents in adolescents with EDs is therefore a concept well worth examining in this population.

### Sense of relational entitlement and pathological concern

From the perspective of attachment theory, both pathological concern and imbalanced sense of entitlement develop early in life and can be understood as defensive relational strategies that develop in response to the absence of available and responsive primary attachment figures. This means that the capacity for mutual need satisfaction that characterizes healthy and mutually satisfying connections fails to develop in a healthy way. Research examining both pathological concern and sense of relational entitlement has shown that people with an imbalanced sense of entitlement have trouble exhibiting concern for both self and other because the mutuality necessary for a healthy sense of concern is lacking [43, 51–54]. A recent study observed a significant association between disordered eating, imbalanced sense of entitlement and pathological concern in a community sample of adult women [53, 54]. Sense of entitlement and pathological concern explained some of the dissatisfaction that women with disordered eating experienced in their romantic relationships. Since sense of relational entitlement and pathological entitlement are hypothesized to develop during infancy, an association with AN/AAN in the current study would raise the possibility that they may be risk factors for this disorder—and perhaps other psychopathologies. However, since the current study is cross-sectional in design, an association would not rule out the possibility that AN/AAN also creates or exacerbates relational imbalance.

Both exaggerated and restricted forms of relational entitlement have been shown to be emotionally maladaptive. Both forms of imbalanced relational entitlement have been found to be connected with negative mood, distress, depression, loneliness, social anxiety, and a lack of life satisfaction [56]. Pathological concern has similarly been shown to be positively and significantly associated with emotional distress and negative emotions, and negatively associated with positive emotions, life satisfaction and self-esteem [43]. We propose that the depression and anxiety that are associated with AN/ANN, whether they precede, co-occur with, or result from the disorder, may be intricately connected to the relational variables of sense of relational entitlement and pathological concern. This connection may therefore help explain (i.e., mediate) the association between AN/AAN and emotional distress in the form of depression and anxiety.

This study aimed to explore the connections between EDs (AN/AAN), pathological concern, sense of entitlement toward parents, depression, and anxiety in adolescent girls. We hypothesized that:

1. Inflated and restricted sense of entitlement would be significantly and positively associated with ED pathology, pathological concern, and symptoms of depression and anxiety.
2. AN/AAN participants would report higher levels of restricted and inflated sense of relational entitlement, pathological concern, and symptoms of depression and anxiety than controls.
3. Sense of relational entitlement (inflated and restricted) and pathological concern would mediate the association between group ([A]AN/control) and symptoms of depression.
4. Sense of relational entitlement (inflated and restricted) and pathological concern would mediate the association between group ([A]AN /control) and symptoms of anxiety.
5. For AN/AAN participants, sense of relationship entitlement (inflated and restricted) and pathological concern would explain a large proportion of the variance in ED pathology after controlling for symptoms of depression and anxiety.

## Methods

### Participants

Participants were 185 teenage girls aged 12–18 (*M* = *15.08, SD* = *1.39*), 85 with a diagnosis of AN (n = 65) or AAN (n = 20). They were recruited between September 2020 and November 2021 through the ED outpatient clinic at Schneider Children's Medical Center in Petach Tikva, Israel, and diagnosed upon admission by clinical psychologists who specialized in EDs. The control group comprised 100 teenage girls recruited via social networks. A total of 119 girls began to complete questionnaires, however 19 were not included in the analyses because they dropped out without completing their participation in the study. No significant differences were observed between completers and non-completers. All questionnaires were completed by all girls in the clinical group, since this a requirement during intake. Control participants responded online to questions about eating disorder symptoms based on the Mini International Neuropsychiatric Interview for Children and Adolescents (MINI-KID; [44]. Individuals whose responses were indicative or anorexia nervosa or bulimia nervosa (n = 4) in accordance with the guidelines presented by Sheehan et al. [44] were offered therapy and excluded from analyses. We examined BMI percentile adjusted for age. As expected, the mean of the AN group (*M* = *26.17, S.D.* = *23.76*) was significantly lower than the mean of the control participants (*M* = *46.51, SD* = 26.92*)* and the AAN participants (*M* = *58.24, SD* = *30.0,F* = 16.96 *p* < 0.001*).* Since there were no significant differences between the AN and AAN participants for any of the study variables, they were combined into a single AN/AAN group. Participants with AN/AAN were significantly younger than controls (*M* = *14.97, S.D.* = *1.38* vs* M* = *15.85, S.D.* = *1.68*,*t* = *3.93, p* < *0.001)*, so age was included as a covariate in analyses. Most participants were secular (n = 109), 40 were traditional and 36 religious, with no significant between-group (control vs AN/AAN) difference in level of religiosity.

#### Instruments

ED pathology was assessed using the Drive for Thinness, Body Dissatisfaction and Bulimia subscales of the Eating Disorders Inventory III (EDI-III, Garner, 24, a 91-item questionnaire widely used to assess symptoms and psychological features of EDs. It was completed by AN/AAN but not control participants. In this study, an “eating disorder risk” factor (EDI-III-EDR) including Drive for Thinness (seven items, e.g. “I eat sweets and carbohydrates without feeling guilty”), Body Dissatisfaction (eight items, e.g. “I think that my stomach is too big”) and Bulimia (eight items, e.g. “I have the thought of trying to vomit in order to lose weight”) was used in analyses, as recommended by Garner 24. Responses were recorded on a scale between 1 (never) and 6 (always) so that the range of total scores is 15–90. A Hebrew translation [37] yielded Cronbach's alphas of α = 0.85 (Drive for Thinness), α = 0.94 (Body Dissatisfaction) and α = 0.78 (Bulimia). For this sample Cronbach’s alphas were α = 0.90 (Drive for Thinness), α = 0.91 (Body Dissatisfaction) α = 0.80 (Bulimia) and α = 0.83 for the composite scale.

Sense of entitlement towards parents was measured using the Sense of Relational Entitlement among adolescents toward their parents scale (SRE-ap; [51]. The SRE-ap used in this study contained 15 items assessing two types of entitlement: Restricted, or subjective unworthiness vis-à-vis parents, e.g. “I feel my parents deserve more than they get from me”,and inflated, or beliefs that parents should fulfill all needs and frustration when they do not, e.g. “I feel I do not deserve to be frustrated by my parents”. Responses were recorded on a scale between 1 (not true at all) and 5 (very true), so that the range of total scores was 15–75. The SRE-ap, originally written in Hebrew, had alpha Cronbach’s alphas of α = 0.91 (restricted) and α = 0.90 (inflated) in this study.

Pathological concern towards parents was measured using the 18-item Pathological Concern Questionnaire (PCQ; [43] that was adapted to target parents specifically. The PCQ asks about (1) repression and denial of one’s own needs, e.g. “I appear to be independent and resilient, and I find it difficult to rely emotionally on my parents”,and (2) excessive investment in satisfying others’ needs, e.g. “I avoid conflict, anger and direct expression of displeasure in order to preserve contact with my parents”. Responses were recorded on a 7-point scale between 1 (completely disagree) and 7 (completely agree), so that the range of total scores was 18–126. The PCQ, written originally in Hebrew, is reliable and valid [43] and Cronbach's alpha in this study was *α* = 0.91.

Symptoms of depression were assessed via the 33-item child version of the Mood and Feelings Questionnaire (MFQ; [18]. MFQ items cover affective, cognitive, melancholic, vegetative, and suicidal aspects of depression during the past two weeks. A sample item is “I felt so tired I just sat around and did nothing”. Responses are noted on a 3-point scale (0 = not true,1 = sometimes; 2 = true), so that the range of total scores is 0–66. The total optimal MFQ cut‐off score for a diagnosis of major depression in samples of adolescent psychiatric outpatients is 27 [47, 60]. The questionnaire has good psychometric properties in English [47] and Hebrew [13] and Cronbach’s alpha in this study was *α* = 0.95.

Anxiety levels were measured by the 41-item Screen for Child Anxiety Related Emotional Disorder (SCARED; [9]. Items inquire about somatic anxiety/panic, general anxiety, separation anxiety, social phobia and school phobia. A sample item is “When I feel frightened, it is hard for me to breathe”. Responses are noted on a 3-point scale between 0 (almost never) and 2 (very often), so that the range of total scores is 0–82. The SCARED has good psychometric properties (Hale et al., 29. A total score of 25 on the SCARED is the optimal cutoff point that maximizes sensitivity and specificity for clinical anxiety disorders [9]. In this study, a Hebrew translation previously used in research [8] and Cronbach’s alpha was 0.93.

#### Procedure

The study received approval via the Schneider Children's Medical Center Helsinki Committee. Parents were approached, via the hospital outpatient clinic (AN/AAN group) or via the social media (control group), and informed about the study. After they provided informed consent for their adolescent daughters to participate in the study, the adolescents received a full explanation about the study via an email from the outpatient clinical staff and provided informed consent. Participants then completed questionnaires online, in their own time and without supervision from parents or hospital staff, either via the hospital’s web application (“Redcap”) or via Qualtrics (www.qualtrics.com).

#### Data analysis

Data was analyzed using SPSS 22. Pearson correlations were calculated to examine associations between variables. A two-way between-subject (group: control, AN/AAN) multivariate ANCOVA, with 5 dependent variables (sense of relational entitlement [inflated and restricted; SRE-ap], pathological concern [PCQ], depression [MFQ) and anxiety [SCARED]), was conducted to compare scores of AN participants and controls. Age was entered as a covariate. Hayes’ [32] Macro Process via bootstrapping method was used to assess sense of relational entitlement and pathological concern as mediators of the association between group status (ED/control) and depression and anxiety symptoms. Hierarchical Regression analysis assessed explained variance, with ED pathology (EDI-III-EDR) as the dependent variable. In Step 1, symptoms of depression (MFQ) and anxiety (SCARED) were entered and in Step 2, pathological concern, inflated and restricted sense of entitlement (SRE-ap). An a priori power analysis including all study variables and pertaining predicted an effect size of 0.3 with sample size = 185. Alpha error probability was 0.03 and power was 0.97 [22]. The power analysis pertained to comparisons between the control and the AN/AAN group, since this was the main objective of the study. The choice of 0.3 represented the smallest effect size feasible, providing us with a conservative estimate. It should be noted that larger effect sizes would still fall within the range supported by our sample size. Since only participants who completed all the questionnaires were included in the analyses, there was no missing data. Age was entered as a covariate in all relevant analyses.

## Results

### Hypothesis 1

*Inflated and restricted sense of entitlement (SRE-ap) would be significantly and positively associated with ED pathology (EDI-III-EDR), pathological concern (PCQ), symptoms of depression (MFQ), and symptoms of anxiety (SCARED).*

Table 1 presents Pearson associations between all study variables. Inflated and restricted sense of entitlement (SRE-ap) were positively and significantly associated with all other study variables, when controlling for age, which was entered as a covariate in all Pearson associations. Eating pathology (EDI-III-EDR) was positively and significantly associated with pathological concern (PCQ), symptoms of depression (MFQ) and anxiety (SCARED). Pathological concern (PCQ) was positively and significantly associated with symptoms of depression (MFQ) and anxiety (SCARED). Symptoms of depression (MFQ) and anxiety (SCARED) were positively and significantly associated.

**Table 1:** Correlations of all study variables, with age entered as a covariate

	Inflated sense of entitlement	Restricted sense of entitlement	ED pathology	Pathological concern	Symptoms of depression	Anxiety
Inflated sense of entitlement (SRE-ap)		.39***	.31**	.45***	.44***	.32***
Restricted sense of entitlement (SRE-ap)			.57***	.65***	.61***	.58***
ED pathology (EDI-III-EDR)				.75***	.72***	.61***
Pathological concern (PCQ)					.74***	.74***
Symptoms of depression (MFQ)						.69***
Mean (SD)	2.68 (.99)	2.37 (1.08)	3.72 (2.01)	4.02 (1.27)	.78 (.66)	.79 (.36)

N = 185 for correlations between all study variables, without ED pathology. N = 85 for correlations with ED pathology; SRE-ap, sense of relational entitlement scale—adolescents toward their parents; EDI-III-EDR, eating disorders risk factor of the eating disorders inventory—III; PCQ, pathological concern questionnaire; MFQ, mood and feelings questionnaire; SCARED, screen for child anxiety related emotional disorders

^*^p < .05; **p < .01; ***p < .001

### Hypothesis 2

*AN/AAN participants would report higher levels of restricted and inflated sense of relational entitlement (SRE-ap), pathological concern (PCR), symptoms of depression (MFQ) and anxiety (SCARED) than controls.*

**Fig. 1:**
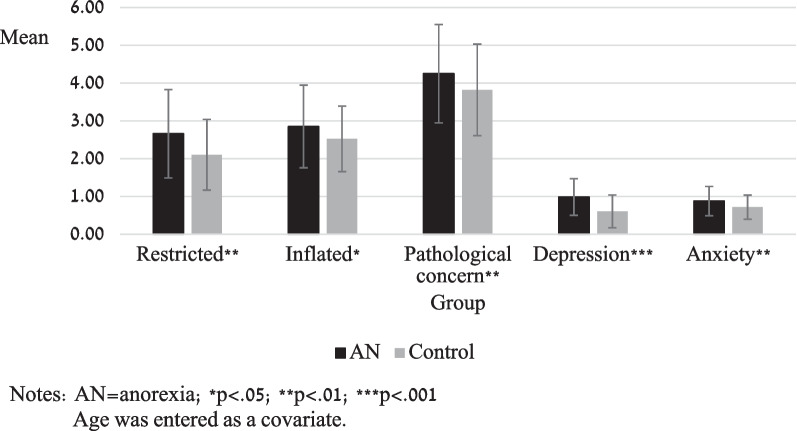
Group differences for sense of entitlement, pathological concern, depression, and anxiety. *Notes* AN = anorexia; *p < .05; **p < .01; ***p < .001. Age was entered as a covariate

A two-way between-subjects (group: control, AN/AAN) multivariate ANCOVA, with 5 dependent variables (sense of relational entitlement [inflated and restricted; SRE-ap], pathological concern [PCQ], depression [MFQ] and anxiety [SCARED]) was conducted, controlling for age. Significant between-group differences were observed (F_(5,182)_ = 7.04, p < 0.001, ηp^2^ = 0.16; see Fig. 1). AN/AAN participants scored significantly higher than controls on restricted (SRE-ap; F_(1,186)_ = 8.33, p = 0.004, ηp^2^ = 0.04) and inflated (SRE-ap; F _(1, 186)_ = 4.22, p = 0.04, ηp^2^ = 0.02) sense of entitlement, pathological concern (PCQ; F_(1, 186)_ = 6.52, p = 0.01, ηp^2^ = 0.03) symptoms of depression (MFQ; F_(1, 186)_ = 30.57, p < 0.001, ηp^2^ = 0.14) and anxiety (SCARED; F_(1, 186)_ = 9.97, p = 0.002, ηp^2^ = 0.05).

### Hypothesis 3

*Sense of relational entitlement (inflated and restricted; SRE-ap) and pathological concern (PCQ) would mediate the association between group ([A]AN/control) and symptoms of depression (MFQ).*

Following Hayes’ [32] Macro Process via bootstrapping method, we used the bias-corrected 95% CI around the indirect effect from 5000 bootstrap re-samples. We accepted the indirect effect as statistically significant if its bias-corrected 95% CI excluded zero.

**Fig. 2:**
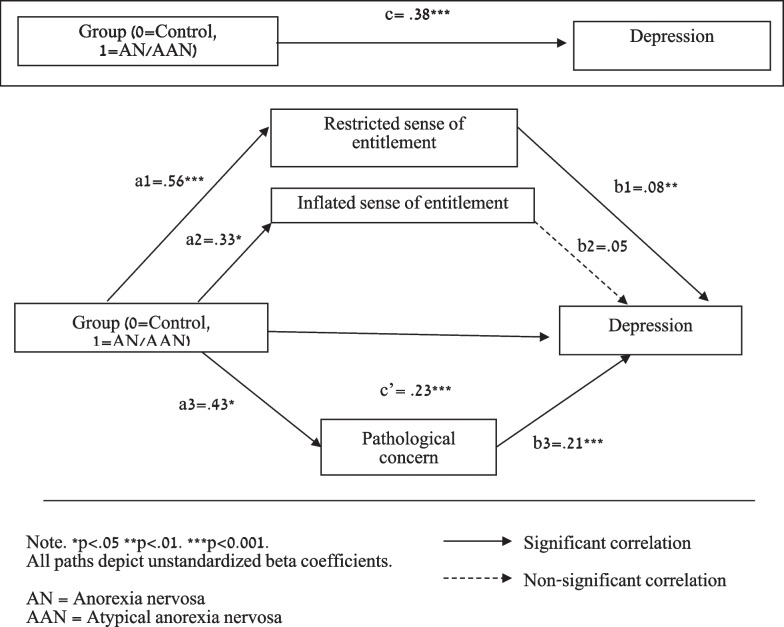
Mediation effect of sense of entitlement and pathological concern on the association between group and depression (n = 185)

Figure 2 shows a significant total effect between Group and symptoms of depression (MFQ). Paths a1 (Group on restricted sense of entitlement), a2 (Group on inflated sense of entitlement [SRE-ap]) and a3 (Group on pathological concern [PCQ]) were statistically significant. Paths b1 (restricted sense of entitlement [SRE-ap] on symptoms of depression [MFQ]) and b3 (pathological concern [PCQ] on symptoms of depression [MFQ]) were both statistically significant, but not path b2 (inflated sense of entitlement [SRE-ap] on symptoms of depression [MFQ]). When the mediating variables were entered, the relationship between Group and symptoms of depression [MFQ] remained statistically significant, indicating partial mediation. In addition, CI 95% fell between 1.11 and 8.45, indicating statistically significant mediation. Restricted sense of entitlement [SRE-ap] and pathological concern [PCR] partially mediated the association between Group and symptoms of depression [MFQ].

### Hypothesis 4

*Sense of relational entitlement (inflated and restricted; SRE-ap) and pathological concern (PCQ) would mediate the association between group * [A]AN*/control) and symptoms of anxiety (SCARED).*

**Fig. 3:**
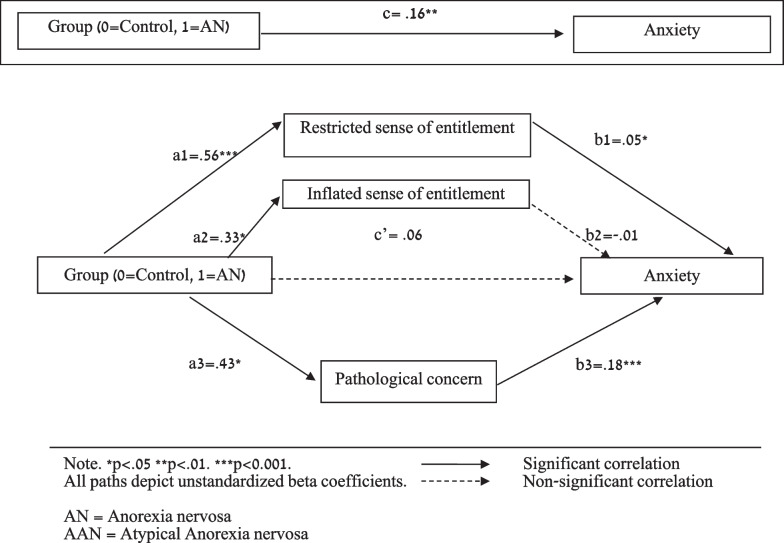
Mediation effect of sense of entitlement and pathological concern on the association between group and anxiety (n = 85)

Figure 3 shows a significant total effect between anxiety symptoms (SCARED) and Group. Paths a1 (Group on restricted sense of entitlement [SRE-ap]), a2 (Group on inflated sense of entitlement [SRE-ap) and a3 (Group on pathological concern [PCQ]) were statistically significant. Paths b1 (restricted sense of entitlement [SRE-ap] on symptoms of anxiety [SCARED]) and b3 (Pathological concern [PCQ] on symptoms of anxiety [SCARED]) were statistically significant, but not path b2 (Inflated sense of entitlement [SRE-ap] on symptoms of anxiety [SCARED]). Finally, when mediating variables were entered, the relationship between Group and symptoms of anxiety [SCARED] was not significant, indicating full mediation, and CI 95% fell between 1.15 and 7.43. Restricted sense of entitlement [SRE-ap] and pathological concern [PCQ] fully mediated the association between Group and symptoms of anxiety [SCARED].

### Hypothesis 5

*For AN/AAN participants, sense of relationship entitlement (inflated and restricted; SRE-ap) and pathological concern (PCQ) would explain a large proportion of the variance in ED pathology (EDI-III-EDR), after controlling for symptoms of depression (MFQ) and anxiety (SCARED).*

To test this hypothesis, we conducted a hierarchical regression analysis with ED pathology (EDI-III-EDR) as the dependent variable (see Table 2). In Step 1, symptoms of depression (MFQ) and anxiety (SCARED) were entered and in Step 2, pathological concern (PCQ), inflated and restricted sense of entitlement (SRE-ap).

**Table 2:** Hierarchical regression analysis with ED pathology (EDI-III-EDR) as dependent variable and SRE-ap and PCQ as independent variables, controlling for MFQ, and SCARED (AN/AAN group, n = 85)

Variables entered	Eating disorder risk (EDI-III-EDR)
*Step 1*	
R^2^ / Adj. R^2^ / F_(*df*)_	.49/.47/_(2,86)_ = 40.49***
Depression (MFQ)	.62***
Anxiety (SCARED)	.10
*Step 2*	
R^2^ / Adj. R^2^ / ΔR/ F_(*df*)_	.57/.54/.08**/_(5,83)_ = 21.64***
Depression (MFQ)	.38**
Anxiety (SCARED)	−.06
Pathological concern (PCQ)	.52***
Restricted sense of entitlement (SRE-ap)	−.04
Inflated sense of entitlement (SRE-ap)	−.06

*p* < .01*; *p* < .05**; *p* < .001***

EDI-III-EDR, eating disorders risk factor of the eating disorders inventory—III; MFQ, mood and feelings questionnaire; SCARED, screen for child anxiety related emotional disorders; PCQ, pathological concern questionnaire; SRE-ap, sense of relational entitlement scale—adolescents toward their parents

For ED pathology (EDI-III-EDR), pathological concern (PCQ) added 11% to the explained variance (when controlling symptoms of depression [MFQ] and anxiety [SCARED]), whereas the contributions of restricted and inflated sense of entitlement (SRE-ap) were insignificant. Symptoms of depression (MFQ), pathological concern (PCQ), but not symptoms of anxiety (SCARED) or sense of relational entitlement (restricted or inflated; SRE-ap), explained a large proportion of the variance in ED risk (EDI-III-EDR), explaining 57% of the variance.

## Discussion

It has been suggested that interpersonal problems [31], such as the tendency towards submissive behavior [17], constitute a core feature of EDs. This study investigated the relational attitudes (sense of relational entitlement and pathological concern) of adolescent girls with and without AN/AAN towards their parents. Adolescents in treatment for AN/AAN reported not only more severe symptoms of depression and anxiety than controls, as expected [41], but higher levels of pathological concern and more imbalanced (both restricted and inflated) sense of entitlement towards their parents. Whereas adolescents with AN/AAN reported a more inflated sense of entitlement than those without, a significantly greater between-group difference was observed for restricted sense of entitlement.

Adolescents with anorexia nervosa and atypical anorexia nervosa therefore seem to have impaired capacity to realistically appraise expectations from their parents, tending to feel over-entitled, yet predominantly under-entitled, to the fulfillment of their needs. Whereas these seemingly opposite tendencies may appear to be mutually exclusive, they are in fact interconnected and should be regarded as two often co-occurring manifestations of imbalanced entitlement [51], just as many people score high on both anxious and avoidant attachment [35]. Restricted entitlement and pathological concern may in fact be driven by a deep sense of injustice at not receiving what one gives to others and longs but dares not ask for. This corresponds to the conceptualization of one's sense of entitlement as an aspect of internal working models of attachment [48].

This finding adds specificity to an accumulating body of research linking imbalanced relational entitlement to other interpersonal difficulties [12, 50, 55]. It also extends the findings of a previous study [51] that when adolescents have either an inflated or restricted sense of relational entitlement towards their parents, they are likely to have emotional problems and low levels of wellbeing, positive mood, self-esteem and life satisfaction.

Our findings further suggest that adolescents with anorexia nervosa or atypical anorexia nervosa tend to focus on their parents’ needs to an extreme degree and at the expense of their own needs, rather than in a balanced and flexible way. Pathological concern also contributed significantly, alongside symptoms of depression, to severity of ED symptoms within the clinical group. These results extend to women with clinical EDs the finding that women in the community with problematic eating attitudes and behaviors tend to ignore self-needs while fulfilling others’ [6, 50, 55]. People with high pathological concern tend to profoundly lack a sense of autonomy, competence and relatedness, have a fragile self-image and be anxious about rejection [26]. The need of adolescents in treatment for anorexia nervosa or atypical anorexia nervosa to over-invest in fulfilling their parents’ needs could therefore perhaps be understood as a compulsive strategy to bolster a shaky sense of worth [52] and avoid abandonment and a sense of isolation [7].

Restricted (but not inflated) sense of entitlement and pathological concern towards parents partially mediated the association between a diagnosis of AN/AAN and symptoms of both depression and anxiety. Since no conclusions about causality can be reached from a cross-sectional design or a mediation analysis, several explanations are possible. AN/AAN may take a heavy toll on emotional health, in part because crucial aspects of relational mutuality have failed to develop. More specifically, a tendency to play down, suppress or deny self-needs vis-à-vis one’s parents and focus on their needs may play a role in the distress experienced by teenage girls with anorexia nervosa or atypical anorexia nervosa. Restricted sense of entitlement and pathological concern influence may also predispose individuals for psychopathology in general and be a risk factor for AN, AAN, depression, anxiety, and perhaps other forms of psychopathology. Finally, anorexia nervosa and atypical anorexia nervosa may lead to, or exacerbate a tendency to deny self needs and focus on others’. One can hardly conceive of a more extreme form of need denial than severe restriction of food intake. A lack of need fulfillment is a basic characteristic of anorexia nervosa and atypical anorexia nervosa and interpersonal ramifications may include restricted sense of relational entitlement and pathological concern.

As hypothesized, correlations between all study variables were significant. In particular, correlational results underscore previous findings that pathological concern and restricted sense of entitlement are associated with symptoms of anxiety and depression in young adults [43, 51]. Since the parent-adolescent relationship has significant implications for adolescents’ ongoing well-being and emotional adjustment [46, 51], adolescents' expectations from their parents are central to their own psychological functioning. We clearly found restricted sense of entitlement and pathological concern towards their parents to be characteristics of girls with AN/AAN. These characteristics involve relinquishing explicit expectations to be cared for and responded to in satisfying ways, a possible maladaptive defensive strategy against disappointment and psychic pain [43, 56], and excessive caring for others, a possible way to avoid connecting to vulnerability and neediness [7].

Another consequence of these imbalanced relational attitudes may be trouble seeking and receiving help to relieve psychological and behavioral distress. Family members, friends and therapists should be aware of these relational obstacles and challenge them whenever possible. Inflated and restricted sense of entitlement and pathological caring should be addressed in therapy for AN and AAN in addition to issues more directly connected with eating and body image problems. It may be complex for teens with EDs to collaborate within a treatment model such as Family Based Therapy (FBT; [34], in which they are expected to accept help, i.e., need fulfillment, from their parents as the basic mechanism for change. Adolescents with an inflated sense of entitlement may tend to experience extreme frustration and a sense of injustice when they feel misunderstood or inadequately supported by their parents. Adolescents with a restricted sense of entitlement and/or high pathological concern may struggle to be helped at all. FBT therapists should be aware of these relational mechanisms when assessing and helping parents to help their children re-learn to eat. On the other hand, improvement and a teen’s sense of having partnered with his/her/their parents to recover from a serious disorder may bring about subtle or even dramatic change for the better in sense of relational entitlement and pathological concern vis-à-vis parents. Change in these variables over time could be assessed in research protocols evaluating outcomes in FBT and other treatment models for AN and AAN. This study is not without limitations. Participants were Jewish, Israeli teenage girls so that results cannot be generalized to other ages, genders or cultures. The clinical sample included teens hospitalized for treatment of anorexia nervosa or atypical anorexia nervoa, so the role of relational attitudes should be examined in relation to other EDs. Future research should examine the role of relational variables in people with other EDs and at other levels of treatment. Relational attitudes were self-reported and not observed in real-life situations. They were also limited to the specific relational context of the parent-adolescent connection. Information on the diagnosis of comorbid disorders was not available and only EDs were screened for in the control group. A measure of ED symptoms was not administered to the control participants, which would have allowed the examination of the relationships between constructs across a fuller range of ED pathology. The study design was cross-sectional, and therefore no conclusions about chronology can be reached. In addition, dependence and resentment that often results from being hospitalized and treated for an ego-syntonic disorder may have contributed to imbalance in the relationship between participants with anorexia nervosa and atypical anorexia nervosa and their parents.

Future research should therefore investigate relational attitudes using longitudinal designs to examine their development over time in relation to parent–child and other relationships, and symptoms of general and ED pathology. It should also be examined whether the relational attitudes of people with EDs become more balanced as recovery progresses. This possibility is in line with Hartmann et al.’s [31] finding that over the course of treatment, the interpersonal patterns of patients with EDs changed significantly. Social connection as a component of recovery from EDs has been advocated by Bachner-Melman et al. 4, 5. Addressing relational issues such as sense of entitlement and pathological altruism in therapy may therefore be helpful in promoting recovery from anorexia nervosa and atypical anorexia nervosa, beyond ED symptoms.

## Conclusions

Relational difficulties experienced by people with anorexia nervosa and atypical anorexia nervosa seem intricately intertwined with their eating disorder, depression, and anxiety symptoms. Their tendency to over-focus on their parents’ needs at the expense of their own may take a toll on their emotional and social health. Relational attitudes may negatively affect help-seeking. Family members, friends and therapists should be aware of these relational obstacles and challenge them whenever possible.

## Data Availability

The datasets used and/or analysed during the current study are available from the corresponding author on reasonable request.

## Abbreviations

AN: Anorexia nervosa
AAN: Atypical anorexia nervosa
BMI: Body mass index
EDs: Eating disorders
SRE-ap: Sense of relational entitlement among adolescents toward their parents’ scale
EDI-III: Eating disorders inventory III
EDI-III-EDR: Eating disorder risk factor
PCQ: Pathological concern questionnaire (PCQ)
MFQ: Mood and feelings questionnaire
SCARED: Screen for child anxiety related emotional disorder
